# Transitional Nerve: A New and Original Classification of a Peripheral Nerve Supported by the Nature of the Accessory Nerve (CN XI)

**DOI:** 10.1155/2010/476018

**Published:** 2011-01-13

**Authors:** Brion Benninger, Jonathan McNeil

**Affiliations:** ^1^Departments of Surgery, Oral Maxillofacial Surgery and Integrative Biosciences, Schools of Medicine and Dentistry, Oregon Health and Science University, 611 SW Campus, Room 713, Portland, OR 97239, USA; ^2^Department of Integrative Biosciences, School of Dentistry, Oregon Health and Science University, Portland, OR 97239, USA

## Abstract

Classically, the accessory nerve is described as having a cranial and a spinal root. Textbooks are inconsistent with regard to the modality of the spinal root of the accessory nerve. Some authors report the spinal root as general somatic efferent (GSE), while others list a special visceral efferent (SVE) modality. We investigated the comparative, anatomical, embryological, and molecular literature to determine which modality of the accessory nerve was accurate and why a discrepancy exists. We traced the origin of the incongruity to the writings of early comparative anatomists who believed the accessory nerve was either branchial or somatic depending on the origin of its target musculature. Both theories were supported entirely by empirical observations of anatomical and embryological dissections. We find ample evidence including very recent molecular experiments to show the cranial and spinal root are separate entities. Furthermore, we determined the modality of the spinal root is neither GSE or SVE, but a unique peripheral nerve with a distinct modality. We propose a new classification of the accessory nerve as a transitional nerve, which demonstrates characteristics of both spinal and cranial nerves.

## 1. Introduction

**Figure 1:**
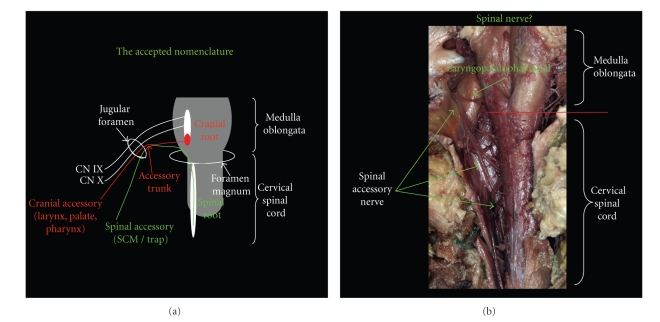
Cranial root of accessory exits from the caudal region of the nucleus ambiguus. The spinal root of the accessory originates within cervical spinal cord and ascends through the foramen magnum. Cranial nerves IX (not pictured), X, and both roots of the accessory exit the jugular foramen. Cranial accessory joins vagus at or beyond the jugular foramen prior to innervating musculature of the palate, pharynx, and larynx. The cranial root of the accessory nerve can be seen passing in close proximity to the spinal accessory and vagus nerves. Purely empirical observations could easily lead to misinterpretation of the cranial root of the accessory nerve. View of cadaver dissection demonstrating the extent of the spinal accessory nerve located within the cervical region of the spinal cord. Also highlighted is the laryngopalatopharngeal (currently cranial accessory) nerve.

 The classification and functional role of the human accessory nerve has been a topic of interest among anatomists dating back to Sir Thomas Willis. Contemporary anatomical texts universally describe the accessory nerve as having two separate components, one from the spinal cord and the other from the brainstem. The spinal accessory is formed from several rootlets, which emerge from the elongated nucleus between C1 and C7. The rootlets join together forming the spinal root of the accessory and ascend through the foramen magnum, where they reportedly join briefly with the cranial root of the accessory to form the accessory nerve *trunk* prior to exiting the skull with the glossopharyngeal and vagus nerves via the jugular foramen. After exiting the skull, the accessory nerve trunk splits into two rami (internal and external). The fibers from the cranial accessory branch or internal ramus, join the vagus nerve branches that contribute to form the pharyngeal plexus and are thought to innervate palatal, pharynx, and larynx muscles. Palate muscles include levator veli palatini, palatoglossus, palatopharyngeus, and musculus uvulae. Pharynx muscles include superior, middle, and inferior constrictors. Other cranial or internal ramus fibers join the recurrent laryngeal branch of the vagus to aid innervating larynx muscles, thyroarytenoid and lateral cricoarytenoid. The spinal accessory branch or external ramus goes on to innervate the sternocleidomastoid (SCM) and trapezius muscles (Figure 1). Most texts list the modality of both the cranial/internal and spinal/external branches or rami as special visceral efferent (SVE), indicating the musculature is derived from the branchial arches [1–12]. However, a few recent texts now describe the spinal root of the accessory as general somatic efferent (GSE), indicating the SCM and trapezius are derived from somites [13, 14]. The discrepancy between the classification and modalities of the two branches of the accessory nerve has yet to be completely resolved in the literature. The authors of this paper have conducted an investigation of the anatomical literature pertaining to the accessory nerve in order to resolve misunderstandings surrounding the relationship between the spinal and cranial roots of the accessory *nerve*, the modality of the spinal accessory nerve, and the embryology of *its target organs*, the SCM/Trapezius complex. After clarifying the misconception of the accessory nerve, we provide a phylogenetic explanation for the development of the spinal accessory nerve based on recent studies in comparative anatomy.

## 2. History of the Nerves of the Brain

Galen (129–210), the early Greek physician, was the first to differentiate between nerves, ligaments, and tendons [15]. “When we say “nerve” we only mean that which springs from the brain or the spinal marrow…” [15]. The original Greek wording used by Galen, “Enkephalon” or literally brain is used to describe the nerves originating within the brain or brainstem [15]. Our modern day terminology has replaced Galen's original wording with the term “cranial” nerve; however, it is clear from the work of Galen that the distinguishing characteristic was not that the nerves passed out of the skull, but rather they originated within the brain or brainstem as opposed to the spinal marrow (cord). Staying consistent with Galen's original wording, the authors of this paper are in favor of using the term encephalic nerves when referring to the nerves that find origin within the brain or brainstem. Although this may seem like a pragmatic argument, it has considerable importance in our present discussion of the accessory nerve.

In addition to aptly distinguishing between encephalic and spinal nerves, Galen produced one of the first written attempts at counting the encephalic nerves. In “On anatomy of nerves,” Galen identifies ten of the encephalic nerves and organizes them into seven pairs [15, 16]. Galen is the first to identify the accessory nerve, which he includes with the vagus n. and glossopharyngeal n. as his sixth pair. Although he goes little beyond mentioning the apparent innervation into the “muscle of the scapula,” his accomplishment was significant and remained unchallenged for nearly 1500 years [15].

In 1543, Vesalius, and in 1561 Fallopius, produced their own respective treatises on human anatomy, but they did little to challenge Galen's seven pair classification of the encephalic nerves [16]. Finally, in 1664, Sir Thomas Willis disputed the deeply rooted Greek dogma by ascribing a physiological and functional role to the encephalic nerves in his celebrated *Cerebri Anatome* [17]. In Willis' manuscript, 10 pairs of cranial nerves are described. The first six remain in agreement with literature to date: olfactory n. (I), optic n. (II), oculomotor n. (III), trochlear n. (IV), trigeminal n. (V), and abducens n. (VI). His seventh pair groups the facial n. (VII) with vestibulocochlear n. (VIII). The eighth pair arranges the glossopharyngeal n. (IX) with vagus n. (X). The ninth pair consists of the hypoglossal n. (XII), and finally, the tenth pair refers to C1, which Willis includes with some hesitation [17, 18]. Willis, like Galen, emphasizes that the encephalic nerves find origin within the brain, as opposed to the spinal nerves, which begin in the spinal marrow (cord). In spite of the apparent agreement on the distinction between encephalic and spinal nerves, Willis separates himself from earlier anatomists by removing the accessory nerve from his pairings of encephalic nerves. Willis reasons that the accessory nerve is an irregular spinal nerve due to its origin being entirely within the spinal marrow. Willis argues that if the accessory nerve were a typical spinal nerve, then it would take a direct course to its target muscles. Instead, Willis points out that in humans, the accessory nerve begins at the sixth or seventh vertebrae and ascends into the skull, where it is joined by the vagus n. prior to exiting the jugular foramen and begins innervating its respective muscles [17]. Willis speculates and states that the conspicuous communication between the accessory and vagus nerves explains why some movements of the head and neck appear to be involuntary, “for almost all living creatures do not only turn about their necks at any noise to behold whatever might cause fear, but they being any ways affrighted in the twinkling of an eye fly away, their feet, wings, fins, or other parts answerable to them, being set into a rapid motion” [17]. Although Willis used only empirical evidence to support his intuitive view of the accessory nerve, he was accurate on many accounts. 

In 1778, the accessory nerve was again classified as a cranial nerve, this time by Soemmerring [19]. Soemmerring's twelve nerve classification differs slightly from Willis by ungrouping VII/VIII and IX/X and excluding C1 [16, 20]. The major difference between Willis and Soemmerring's classifications rests in Soemmering's inclusion of the accessory nerve as cranial nerve XI. From a location standpoint, it does not make sense that the accessory nerve would be listed as the eleventh cranial nerve when its nucleus and nerve begin more caudally than those of the hypoglossal or XII nerve. Soemmerring is credited for our current classification of the cranial nerves; however, minor alterations have occurred. The most important change for our present discussion is the addition of a cranial/bulbar root of the accessory nerve. The cranial/bulbar root can be traced to Fredrici Arnold whom published a series of elaborately drawn anatomical plates depicting two components to the accessory nerve [21]. Although Arnold does not describe the two components in detail, he clearly depicts them well enough that Henry Gray (1858) references the work in his first edition of Gray's *Anatomy, Descriptive and Surgical* [22]. All anatomical texts published after the release of Gray's highly regarded text, describe two components of the accessory nerve. The author of this paper believes that the structure known as the cranial root of the accessory nerve should not be included as part of the accessory nerve and should be renamed. Furthermore, we intend to demonstrate why Thomas Willis was correct in his reasoning that the accessory nerve is NOT an encephalic nerve and should be regarded as a unique peripheral nerve.

## 3. The Cranial Root of the Accessory Nerve

The cranial root of the accessory nerve has been accepted universally amongst anatomical texts, yet the last three centuries of comparative anatomy has strongly refuted its validity as a component of the accessory nerve [23]. Sir Thomas Willis, one of the earliest comparative anatomists, observed the accessory nerve in various species leading him to conclude, the nerve “…is found constantly, not only in man and four-footed beasts, but also in fowls and fishes” [17]. Willis makes no mention of a second component of the accessory nerve, but he does state that the fibers of the accessory n. join briefly with those of the vagus n. prior to passing from the skull. Willis' failure to include the so-called cranial root of the accessory was not likely an oversight, but an indication that he considered these fibers as part of the vagus nerve. Willis' notion that only the spinal portion of the accessory represents the true accessory proper is backed by modern comparative anatomy, especially the work of renowned Dutch neuroanatomist Kappers [23]. After a thorough investigation of the accessory nerve, Kappers concludes the “cranial” root of the accessory should be regarded as a caudal portion of the vagus nerve. Kapper's postulations have been further supported by more recent comparative studies using retrograde labeling of axons in several different species [24–30]. The question of whether the “cranial” root should be regarded as the caudal-most fibers of the vagus or as a separate entity is still debatable. At least one team, Szekely and Matesz [28], provides evidence in the Sand Lizard that the motoneurons of the so-called cranial accessory differed in both size and location from those of the nucleus ambiguus of the vagus n., thereby indicating the “cranial accessory” is an independent structure altogether.

In 2002, Lachman et al. performed meticulous human dissections of the caudal posterior medullary rootlets (CPMR) aka “cranial accessory” rootlets [31]. In 100% of the cases investigated, Lachman et al. found the CPMR failed to join the spinal root of the accessory nerve, and instead merged with other vagal rootlets to form the superior ganglion of the vagus nerve. These findings are consistent with the author's observations (Figure 1). Only one published investigation was found by Wiles et al. that argues the existence of the so-called cranial root of the accessory nerve [32]. In 12 embalmed cadavers, Wiles et al. found 45% of the time a cranial root of the accessory nerve was present; however, they concede several limitations to their own study. Not only must one appreciate the complexity of the jugular foramen and surrounding structures, but also, the differences between fresh versus embalmed cadaveric tissue. This paper's authors suggest that many of the inconsistencies between the Lachman et al. and Wiles et al. studies can be attributed to the state of tissue at the time of dissection and the method for preserving the integrity of the structures within the jugular foramen. 

The most convincing evidence for the disjunction between the cranial and spinal roots of the accessory nerve does not come from anatomical observations, but rather from molecular investigations into the development of the nervous system. Development of the nervous system is regulated in part by the expression of highly conserved DNA sequences known as homeobox (HOX) genes [33–35]. Homeobox genes are responsible for producing various transcription factors that interact with mediators to produce the various classes of neurons [34, 35]. Recent investigations by Pabst et al. have identified the Nkx2.9 homeobox gene as a key regulator in the development of the spinal accessory nerve [36, 37]. By creating a strain of Nkx2.9 knockout mice, investigators were able to show that the inhibition of the Nkx2.9 gene produced mice that lacked a fully developed spinal accessory nerve. Interestingly, the “cranial” accessory developed normally, strongly indicating a disjunction between the two nerves [37–39]. Although the Nkx2.9 knockout embryos showed evidence of a spinal accessory nucleus, the nerve failed to exit at the lateral exit point (LEP). Therefore, it is logical to assume that a number of other homeobox transcription factors are responsible for upstream and downstream development of the spinal accessory nerve. Dillon (2005) found that Gli2 is essential for the formation of spinal accessory motoneuron cell bodies, while Netrin-1 and DCC have a role in axonal growth between cell body and LEP [39]. Although each individual nerve likely expresses a slightly different combination of transcription factors, the potential to begin classifying the nervous system by the specific genes expressed may soon exist [35]. A molecular classification would allow us to better highlight the similarities between particular nerves while overcoming the shortcomings of the current physiological modalities approach. Nevertheless, by exploiting the anatomical, comparative, and molecular differences between the “cranial” and spinal roots of the accessory n., there is little doubt that these two structures are unique entities. Whether we should consider the “cranial” root as a caudal portion of the nucleus ambiguus or an independent structure is still debatable. It is the author's view that the “cranial” root of the accessory should be regarded as the laryngopalatopharyngeal motor nerve and be the sole representation of the eleventh cranial nerve in the current cranial nerve classification. The remaining portion of this paper will focus on the spinal root of the accessory, which we maintain is the accessory nerve proper.

## 4. The Accessory Nerve Proper

Much of the controversy surrounding the accessory nerve proper is focused on its unique morphology and current alleged modalities. Early comparative anatomists have argued two plausible theories (SVE & GSE) explaining the puzzling composition of the accessory nerve in higher vertebrates. The Special Visceral Efferent (SVE) theory, popularized by Ariens Kappers, suggests the accessory nerve in early vertebrates finds origin within the caudal aspect of the vagal nucleus [23]. As phylogeny progresses, the nucleus of the accessory nerve migrates caudally, eventually becoming an independent structure within the cervical spinal cord. Supporters of the SVE theory argue that if the accessory nerve originates as a caudal portion of the vagus n., then its muscles of origin are presumably derived from the branchial arches; thus, the nerve should have a special visceral efferent modality [23, 40]. The General Somatic Efferent (GSE) theory, proposed by J. L. Addens, argues the accessory nerve is an abnormal spinal nerve and its musculature is somatic in nature, characterizing the nerve as GSE [41]. Unfortunately, both theories proposed for the origin of the accessory nerve were prior to the advent of definitive neurotracing techniques. Furthermore, the precise embryological origins of the SCM and trapezius have only recently been elucidated, allowing us to revisit the debate with a modern perspective. 

Early embryologists believed the striated musculature of the head and neck was derived from the branchial arches [23, 40, 42]. Unfortunately, the presumption of many early anatomists was at times inaccurate. Recent investigations have shown that striated musculature originates from paraxial mesoderm [43–48]. However, there are distinct differences in the behavior of head mesoderm compared to the trunk mesoderm. Below the neck, paraxial mesoderm condenses and epithelializes into somites, a process that is largely regulated by the hairy gene [49]. Each somite gives rise to a particular myotome, dermatome, and sclerotome. Myotomes form the striated muscle for a particular segment of the trunk. Dermatomes are responsible for the formation of the dermis in a particular segment, while sclerotomes develop into the vertebrae and their associated intervertebral disc. In addition, the somite will give rise to angioblasts and hemangioblasts responsible for the vasculature belonging to the tissue developing from a particular segment [47–51]. Finally, bone and connective tissue of the trunk are derived from mesenchyme, which is also a derivative of mesodermal cells [50]. Thus, the embryonic mesoderm is entirely responsible for producing the musculoskeleton and associated components below the neck.

Development of the musculoskeletal components of the head do not follow the strict mesodermal origins observed in the trunk. Striated musculature of the head does not develop from organized mesodermal somites as observed in the trunk region. Instead, loosely organized masses of lateral mesoderm form regions referred to by some authors as somitomeres [52]. Somitomeres are believed to play a role in the segmentation of the vertebrate head; however, this topic has recently been reviewed and is not wholly agreed upon [47, 48]. Regardless, the loosely organized paraxial mesoderm (somitomeres) does contribute to the skeletal muscle of the head, but relies heavily on interactions with neural crest cells. Neural crest cells migrate throughout the body and play a major role in the development of the peripheral nervous system as well as many other components. Recent investigations in embryology have highlighted the role of neural crest cells in forming mesenchyme, connective tissue and osseous components, associated with the striated muscle of the head [43–48]. The interaction between the two embryonic cell populations, mesoderm and neural crest, creates a remarkable interaction, whereby the myotubes and endothelial cells are mesodermally derived, while the connective tissue, tendons, epimysial, and endomysial are formed from neural crest cells [47, 48]. Thus, the striated muscle of the head is truly of dual origin and calls into question the age-old distinction between GSE and SVE. The striated muscle associated with the branchial arches is not formed entirely from the neural crest cells that give rise to the arches, nor is it formed entirely from mesoderm as observed in trunk musculature.

**Figure 2:**
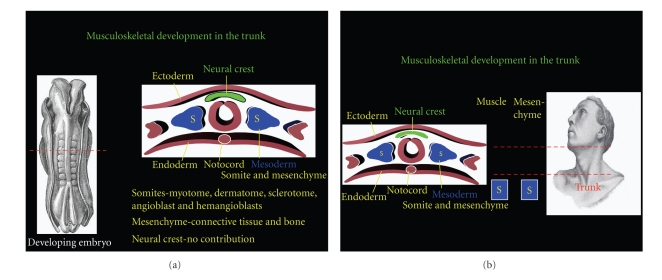
A cross-section of the developing trunk shows the formation of somites from the mesodermal cell population. Each somite forms a sclerotome, myotome, and dermatome. The trunk mesoderm will also form the mesenchyme (connective tissue, ligaments, and osseous attachment) of the trunk, but not the head or neck. Figure 2 reinforces the mesodermal origin of the trunk. Striated muscle arises from the myotome portion of somites, while connective tissue and bone are mesenchymal in origin. The transitional neck region differs by having skeletal muscle derived from somites, while the mesenchymal component is formed from the neural crest.

**Figure 3:**
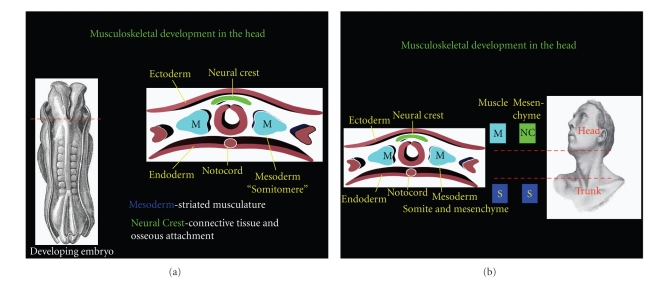
A cross-section through the developing head region shows mesoderm forming somitomeres instead of somites. Somitomeres contribute to the formation of striated muscle in the head, while neural crest cells form the mesenchymal component. A cross-section through the developing embryo head region is seen on the right. In the head, mesoderm does not develop into somites, but remains loosely organized which some authors refer to as “SOMITOMERES”. The mesoderm will still form striated musculature in the head, however; neural crest cells contribute to the connective tissue and the osseous attachment sites.

**Figure 4:**
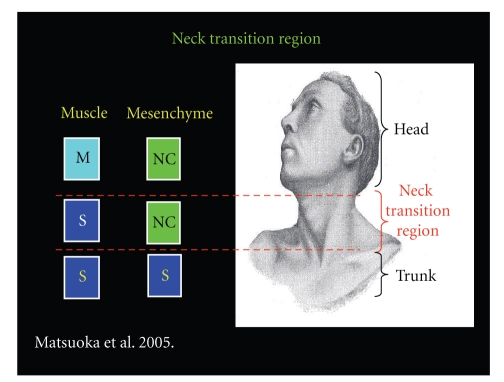
The neck region is a unique region recently highlighted by Matsuoka et al. [53]. The striated muscle in the neck is formed from somites, while the connective tissue and osseous attachment are derived from neural crest cells. Thus, the neck has components of both head and trunk and can be viewed as a transition between the two.

The neck, unlike the head and trunk, has remained relatively ambiguous. Until recently, muscles of the neck (SCM, trapezius, intrinsic laryngeals, external laryngeal, tongue, and occipitocervical muscles) were believed to originate entirely from the more rostral somites [43–48]. An investigation by Matsuoka et al. (2005) challenges the strict somite origin of the neck musculature [53]. Matsuoka et al. contest the widely held ossification model, which maintains bones are either dermal (neural crest derived, e.g., bones of the skull), or endochondral (mesoderm derived, e.g., long bones), depending on where they are located within the developing embryo. Instead, Matsuoka et al. propose a “muscle scaffold model,” arguing that muscular attachment sites determine the cell population of the respective bone regardless of whether dermal or endochondral ossification occur. By tracing cell populations in the neck of mice, Matsuoka et al. make evident the dual origin of neck musculature, especially the SCM and Trapezius, which have specific osseous attachment points and connective tissue formed from neural crest cells similar to head musculature, while the muscle itself and the remaining bone are somite derived [53]. For example, the SCM has tendons that attach to specific bony sites on the mastoid, sternum, and clavicle, which are all neural crest in origin. On the other hand, the remaining portion of the mastoid, sternum, and clavicle are formed from mesodermal components, and the muscle itself is somite derived. Essentially, the neck represents a transitional region between head and body where the classic derivations are not rigorously followed (Figures 2, 3, and 4). The SCM and Trapezius are unique in the sense that they are derived from both neural crest and somites and are innervated by the accessory nerve, which is neither a true cranial nor a true spinal nerve. The authors of this paper do not believe the accessory nerve can be characterized by either GSE or SVE. In reality, the accessory nerve represents parts of both theories and should be regarded as a new category of peripheral nerve, the* Transitional* Nerve (TN).

By applying contemporary embryological and anatomical findings, we can group the efferent peripheral nerves that innervate striated musculature into 3 groups: cranial, spinal, and transitional. Staying consistent with the original definition by Galen, Willis, and others, all cranial nerves have a nucleus of origin within the brain or brainstem. In addition to having a nucleus located in the brain or brainstem, all motor cranial nerves innervate striated musculature that has tendons and attachment sites formed from neural crest cells. The authors propose that cranial nerves can further be grouped into 3 subcategories: cranial somatic efferent with target musculature derived from pre-otic somites (CSE_pr_), (oculomotor (III), troclear (IV), and abducens (VI)); cranial somatic efferent with target musculature derived from postotic somites (CSE_po_) (vagus (X), Laryngopalatopharyngeal motor (XI), and hypoglossal (XII)), and cranial branchial efferent (CBE) (trigeminal (V), facial (VII), glossopharyngeal (IX)), which have targeted musculature arising from somitomeres (nonsomite paraxial mesoderm). Efferent spinal nerves have a nucleus of origin within the spinal cord and innervate musculature that is derived entirely from somites and connective tissue that originates from mesoderm. The authors of this paper maintain a third classification of peripheral nerve, a *transitional somatic efferent* (TSE) nerve, which represents the accessory nerve proper and combines characteristics of both cranial and spinal nerves (Table 1). The accessory nerve is similar to the CBE nerves in that it maintains a lateral exit point and has a cell column in line with the branchial efferent nerves [35]. The branchial link is further supported by its HOX gene expression, which depends on Nkx2.9 a gene that is closely linked to Nkx2.2 expressed by other CBE nerves [39]. Finally, similar to the target musculature of other cranial nerve efferents, the accessory nerve has target musculature that has connective tissue and skeletal attachments derived from neural crest cells [53]. Despite these branchial characteristics, the accessory nerve has a nucleus located within the spinal cord and innervates the SCM and Trapezius, which are derived from cervical somites, similar to a spinal nerve. Its spinal character is further observed in the dual innervation of the SCM and Trapezius by the rostral cervical spinal nerves in combination with the accessory nerve in many vertebrate species. Thus, the authors of this paper propose the SCM and Trapezius are *transitional* muscles, a new category of muscle between the head and neck. Our discussion calls into question the reliability of using the classic modalities of GSE and SVE to describe the motor innervation of the peripheral nerves. The discovery of HOX genes has created an alternative foundation for classifying peripheral nerves. The authors propose combining HOX expression, classic anatomical and modern embryological evidence to create a definitive classification of all peripheral nerves, which will clearly expand on our current distinctions.

## 5. The Transitional Nerve

**Figure 5:**
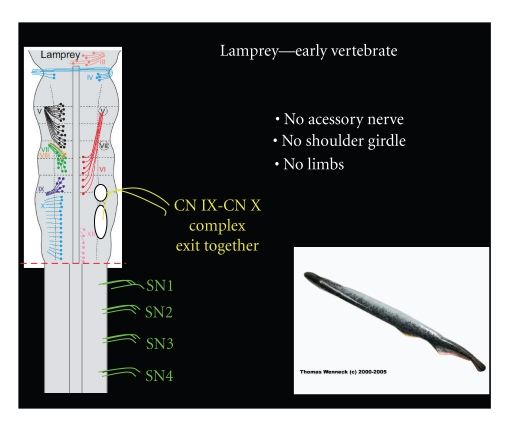
The lamprey lacks an accessory nerve, but has a glossopharyngeal and vagus root that are closely associated.

The lamprey, a limbless eel-like creature, is one of the earliest known vertebrates. Lampreys lack an accessory nerve and the corresponding shoulder girdle [23, 54]. The Cuculalris or homolog of the SCM and trapezius is also absent, thus suggesting that the “neck” region has yet to develop [55]. Additionally, no paired fins are present and the majority of locomotion is accomplished via a dorsal motor fin [23, 55]. The glossopharyngeal and vagus nerves have developed in the lamprey, appearing in a primitive state of specialization and are closely related to each other both in appearance and location of nuclei (Figure 5) [23, 56]. It is important to note that the intestinal ramus of the vagus is also present. This structure which runs caudally along the esophagus and foregut is closely associated with the early appearance of the accessory nerve [56].

**Figure 6:**
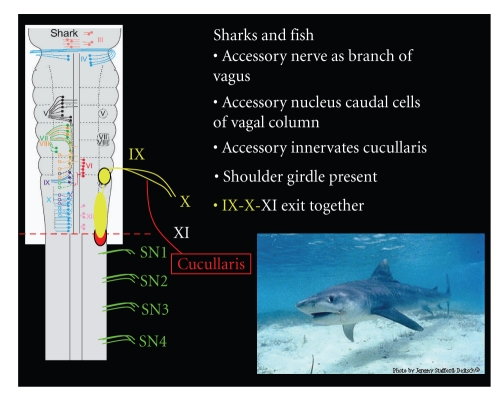
The skate is one of the earliest vertebrates to develop an accessory nerve, which arises from the intestinal branch of the vagus and innervates the Cucullaris (SCM/Trapezius homolog) muscle attaching to the lower branchial arches. The cell bodies contributing axons to the accessory n. in the skate are located in the caudal aspect of the dorsal motor nucleus of the vagus nerve.

The precise rise of the accessory nerve is difficult to ascertain, but its origin can be observed in the next phylogenetic jump in vertebrates, the skates. Skates are cartilaginous fish that have large flattened pectoral fins and can be regarded as a primitive ancestor of sharks [23, 57]. The skate is one of the first species to develop a shoulder girdle, corresponding Cucullaris musculature (Trapezius/SCM homolog), and accessory nerve [23]. The skate was originally thought to have a Cucullaris, represented by three muscular slits: medial, intermediate, and lateral [58–60]. More recent investigations by Sperry and Boord have shown only the lateral slit is innervated wholly by the accessory nerve, while the medial and intermediate receive innervations from spinal nerves 10–14 [60, 61]. The lateral slit consists of a larger superficial part and a smaller deeper part similar to the Cucullaris of the shark. The early Cucullaris of the skate attaches to the shoulder girdle and lower branchial arches and apparently functions in elevation and protraction of the pectoral girdle and a part of the branchial skeleton [60, 61]. The accessory nerve of the skate is composed of axons traveling exclusively within the intestinal ramus of the vagus n. Recall that the intestinal ramus was relatively well formed in the early lamprey (Figure 6) [56, 61]. The motoneurons that supply the accessory n. are present in the caudal aspect of the ventral nucleus of the vagus, thus indicating an undeniable link between early accessory and vagus nerves. The more rostral motoneurons begin at the obex of the medulla and are located ventrolateral to the dorsal motor nucleus of X, while the caudal-most motoneurons are found in the gray spinal matter lateral to the motoneurons of the 3rd/4th ventral spinal roots [61]. Examination of the fibers within the accessory nerve revealed no sensory fibers are distributed with the accessory nerve, indicating the early accessory nerve conveys only efferent motor innervations [61]. 

Sharks, the more evolved cousin of the skate, have a well-developed shoulder girdle and Cucullaris m. that receive innervation from the accessory nerve. The accessory nerve arises again as a branch of the intestinal ramus of the vagus nerve [23, 59]. The vagoaccessorius n. exclusively innervates the cucullaris in at least two species (Alopias and Cynias), while other species (heterodontus, hexanchus, chlamydoselachus, heptanchus, and squalus mitsukurii) receive dual innervations from cervical spinal and vagoaccessorius efferents [23, 59]. Investigations utilizing modern retrograde tracing techniques are lacking in the shark; therefore, the literature should be approached with some skepticism because the complex morphology of the accessory nerve and nucleus make empirical conclusions difficult and often erroneous. Fish present similar problems in the literature as definitive tracing studies are again lacking. Work by Edgeworth [59] supports the contention that in fish, the accessory is closely associated with the vagus nerve leaving the brainstem as the vagoaccessorius complex. In general, the accessory nerve is present in early vertebrates that have developed a shoulder girdle and corresponding Cucullaris musculature [23, 54]. 

**Figure 7:**
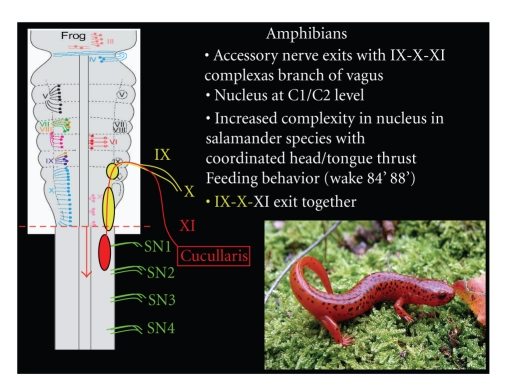
In amphibians, the accessory nerve continues to exit with the glossopharyngeal-vagus complex, however the nucleus has seperated in many species and is located within the spinal column. The accessory nerve innervated the cucullaris musculature which is associated with neck movement and feeding behavior. The accessory nerve is believed to have a universal presence in amphibians. The accessory nerve arises from a nucleus that overlaps portions of the upper cervical nerves. The nerve fibers exit via the IX, X, XI complex.

Amphibians represent the next jump in vertebrate evolution bridging the transition between aquatic and terrestrial species. Recent retrograde neurotrace studies highlight the presence of the accessory nerve in two different species of toad and twenty-two different species of salamander, suggesting its presence is universal amongst amphibians [24, 25, 27, 30, 62]. In salamanders, the accessory nerve exits with the IX, X, and XI complex, while its nucleus is closely associated with the first and second spinal nerves (Figure 7). The feeding behavior in amphibians relies strongly on the interaction between the target muscles innervated by the first and second spinal nerves and accessory nerve. The first spinal nerve of the salamander has only a ventral motor root consisting of 3-4 rootlets which anastomoses with the 2nd spinal nerve containing both motor and sensory modalities. The first and second nerves typically combine to form the ramus hypoglossus innervating muscles associated with the tongue [63]. On the other hand, the accessory nerve is purely motor and innervates the cucullaris and cephalodorsubpharyngeus muscles, which are crucial for both the neck thrust associated with feeding as well as optomotor tracking [64]. The majority of salamanders use a tongue thrust in conjunction with a forward lunge to capture prey. Interestingly, the lineage of slow moving salamanders, Bolitoglossine, do not use a neck thrust motion and instead rely on a longer, quicker tongue to feed. Furthermore, Bolitoglossine salamanders have little escape mechanisms and rely on immobility to escape detection from predators. Not surprisingly, the cellular morphology of the first and second spinal nerves as well as the accessory nerve of Bolitoglossine are underdeveloped compared to species with more aggressive feeding and locomotive potential [62]. In the Bolitoglossine species, the rostral-caudal extent of the accessory nucleus is restricted, being confined to the second spinal nerve, thus suggesting minimal interaction between the accessory nerve and the first spinal nerve controlling the tongue musculature. In all other species investigated by Wake et al., the spinal accessory nucleus extends from the obex of the medulla to the caudal aspect of the third spinal nucleus, thus suggesting a stronger interaction between accessory and upper cervical motoneurons [62]. There is strong evidence that the spinal accessory nerve has an intimate connection with upper spinal nerves facilitating feeding and movement in some species; however, there is still significant variation reflecting some of the ontogenetic changes amongst amphibians.

**Figure 8:**
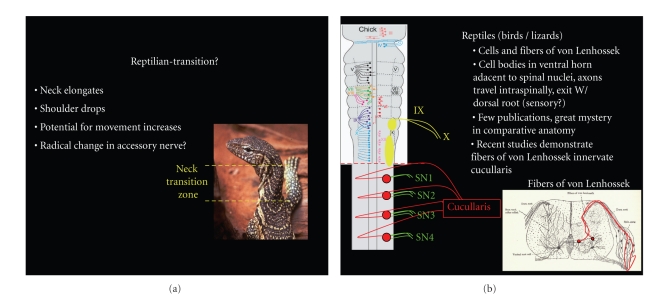
(a) The reptilian body undergoes distinct morphological changes including elongation of the neck and dropping of the shoulder girdles creating the neck transition region. (b) The formation of the neck region may explain the peculiar behavior of the fibers of von Lenhossek, which represent the reptilian spinal accessory nerve. More investigation is necessary to confidently provide a definitive explanation. Cell bodies of the accessory nerve in reptiles are located within the ventral horn of the cervical spinal cord. The axons project intraspinally to exit via the dorsal root. These fibers originally described by von Lenhossek remain poorly understood.

Reptiles as a group are poorly understood in terms of spinal accessory nerve morphology [23, 59, 65, 66]. The spinal accessory nerve has been investigated in snakes, lizards, and birds. The literature encompassing snakes is in agreement that no accessory nerve is present, which is not surprising considering forelimbs, shoulder girdle, and corresponding musculature are also absent [67, 68]. Lizards and birds possess a trapezius/SCM homologue; however, the literature is not always clear on the exact delineation of this musculature and its naming is not always consistent [23, 59, 69–71]. Additional inaccuracies are present due to lack of specificity in staining techniques. In spite of these shortcomings, there are a few well-done studies that indicate the spinal accessory nerve is present in birds and innervates the Cucullaris, which is part of the Complexis or group of “hatching” muscles [69, 71, 72]. Investigations of the Chick indicate the spinal accessory nerve is formed from cell groups located within the ventral horn from levels C2–C4. However, instead of exiting at a point midway between the dorsal and ventral roots as noted in mammals, their axons course through the spinal cord to exit with the dorsal roots of C2–C4 (Figure 8) [69, 71]. The unusual projection of these nervous fibers was first noted by von Lenhossek, and assumed to be the equivalent of the reptilian spinal accessory [23, 71]. The nerve fibers of von Lenhossek remain poorly understood; however, similar phenomena have been reported in lizards, suggesting that these nerve fibers represent the spinal accessory in reptiles or at least a majority of reptilian species [23, 25, 28]. Although more investigations are necessary to draw firm conclusions on the spinal accessory of reptiles, it is important to note the structural changes that occur in the reptilian vertebrate. With the exception of snakes, the reptilian body has developed a distinct neck transition region with a clear elongation of the cervical spinal cord. The morphological changes that occur in reptiles may be associated with the unusual behavior of the spinal accessory nerve (Figure 8).

In mammals, the accessory proper is largely present, but exceptions have been noted in certain orders of ungulates (giraffes, okapi, camels, and lamas) although the literature on these unique species is contradictory [23, 73]. At least one ungulate, the camel, has an accessory proper, which emits as several nervous fibers that do not unite, but rather pass directly to the target muscle as individual fibers. This variation has not been well studied and it is not clear if any similarities are present between the camel and arrangements observed in some reptiles [23]. Beyond the few noted exceptions in ungulates, the accessory proper has been observed in a number of mammals in its normal course, that is taking origin within the upper cervical spinal cord and emitting fibers, which join together prior to passing through the foramen magnum to exit the jugular foramen with the glossopharyngeal and vagus nerves. 

**Figure 9:**
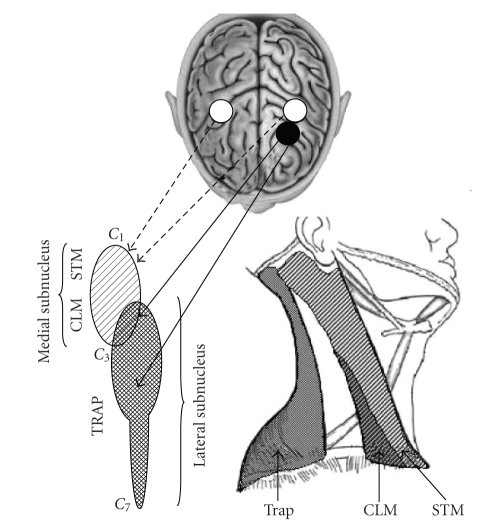
The accessory nucleus is composed of a medial and lateral subnucleus. The sternomastoid receives innervation from the medial subnucleus, which in turn receives bilateral projections from the primary motor cortex. The sternomastoid is thought to be exquisitely involved in oculomotor tracking. The cleidomastoid receives innervation from the caudal part of the medial subnucleus as well as the rostral lateral subnucleus. The trapezius receives innervation from the majority of the lateral subnucleus. The cleidomastoid and trapezius have contralateral cortical representations in the supplementary motor cortex and are believed to play a role in stabilization of the neck as well as locomotion in some species.

Detailed studies of the spinal accessory nucleus and nerve have been performed in a number of mammalian species, especially the rat, cat, monkey, and human [74–81]. Early investigators observed a single “pearl-like” strand of cell bodies that had a caudal limit around C5 [23, 82–84]. More recent investigations provide sound evidence of two distinct spindle-shaped subnuclei [75, 76, 78–81, 85]. In a meticulously investigation of the rat, Krammer et al. (1987) found the medial subnucleus of the accessory proper begins at the medullary/spinal transition zone and extends to around C2 where its neuronal density decreases considerably. By the C3 level, no medial subnucleus neurons appear. The lateral subnucleus of the rat begins at the rostral C2 level and continues caudally to C7 where neuronal density tapers considerably [76]. Interestingly, a number of investigations have found the subnuclei of the accessory proper to be somatotopically organized in higher mammalian vertebrates [76, 77, 79, 80]. The majority of the medial subnucleus innervates the sternomastoid muscle or the sternomastoid portion of the SCM when fused with the cleidomastoid in humans. The cleidomastoid receives innervation from a small caudal area of the medial subnucleus and a small rostral area of the lateral subnucleus, while the trapezius is innervated by the majority of the lateral subnucleus (Figure 9) [76, 77, 79, 80]. One final notable characteristic of the accessory muscle complex is the distinct cortical representation. The sternomastoid muscle differs from the cleidomastoid and trapezius muscles, having a cortical representation in the primary motor cortex near the head and thumb and receiving projections from both cerebral hemispheres [76, 86–88]. On the other hand, the cleidomastoid and trapezius muscles have cortical representation primarily in the supplementary motor cortex and receive projections from contralateral innervation (Figure 9) [86–88]. The accessory nucleus is a fascinating phenomena that closely resembles the nucleus of CN VII which also has a rostral portion receiving bilateral innervations and a caudal element receiving only contralateral fibers [87].

In addition to the distinct nuclear properties, the accessory proper shows peculiar interactions with the rostral cervical nerves, unlike any other nerve in the body. Several investigators have observed various intra and extra dural anastomoses between the accessory proper and the upper cervical nerves. These connections have been documented in a number of species including various sharks, lizards, and more extensively in the rat, cat, monkey, and human [23, 41, 59, 74, 76, 89–96]. Comparative studies have long emphasized the modality of the accessory proper as only motor; however, many investigations have provided some evidence of proprioceptive fibers being conveyed to the accessory nerve via the upper cervical nerves. This remains a topic of debate [23, 76, 89, 93, 94, 97]. Although the origin of proprioceptive input to the SCM and Trapezius remains controversial, EMG and neurotrace studies have demonstrated the dual innervation of the aforementioned muscles by the upper cervical nerves. Typically, the SCM receives efferent motor from C1 and C2, while the trapezius receives contributions from C2, C3, and C4 [98–104]. These observations can be appreciated in patients who have undergone radical neck dissection with complete loss of accessory nerve, but can still retain limited movement of the muscles [105]. 

By looking at the scope of comparative literature regarding the development of the accessory nerve proper, a plausible explanation for the irregular morphology and behavior is apparent. The accessory nerve first makes its appearance in the early cartilaginous fish (skates and sharks) [40, 59]. The accessory nerve develops closely with the branchial arches taking an attachment on the lower arches in many species [23, 40, 59, 76]. Additionally, the nucleus of the accessory nerve originates as cell bodies within the caudal vagal motor column, and the accessory nerve is represented as a branch off of the intestinal ramus of the primitive vagus nerve. As phylogeny progresses, the accessory nuclear complex migrates caudally. This phenomenon is explained by the theory of Neurobiotaxis originally proposed by Kappers [23, 106]. According to Neurobiotaxis, the cell bodies of a particular group of axons will migrate in the direction that they receive the most frequent stimulation. As vertebrates transition from water to land, the cell bodies of the accessory nerve shift from the medulla oblongata to the cervical portion of the spinal cord, which has become the center of their stimulation. The stimuli come from connections with the sensory and motor neurons of the upper cervical nerves as well as higher centers, which control movements of the neck musculature [23]. Several studies have demonstrated the importance of descending pathways responsible for coordinated head and eye movements such as visual tracking, which terminate in the region of the upper cervical spinal cord in the area of the spinal accessory nucleus [23, 107, 108]. This phenomena is evident in ontogenetic examination of salamanders, which display different stages in accessory and spinal nerve development depending on the complexity of feeding behavior and mobility [62, 64]. In reptiles, the shoulder girdles descend and the neck elongates producing a transition zone between head and body [23, 59, 76]. The accessory nerve takes on a unique morphology in reptiles, which likely facilitates increased mobility of the neck and anterior limb locomotion; however, this group of vertebrates has been the focus of few in depth investigations [23, 76]. In mammals, the nuclear complex of the accessory nerve is strikingly different than any spinal nerve, and more closely resembles the somatotopically arranged facial (CN VII) nucleus [76]. The medial subnuclei of many mammals is strongly linked to the Sternomastoid portion of the SCM and receives dual bilateral cortical representation. Furthermore, many of the descending tracts terminate specifically within the medial subnucleus suggesting the Sternomastoid is crucial for oculomotor tracking and likely recruits both right and left sternomastoids simultaneously [76, 87, 88]. The lateral subnucleus receives unihemispheric contralateral innervation and projects axons to the cleidomastoid and trapezius, muscles that developed primarily for locomotion in quadrapedals and likely offer increased stability to the head and neck region. Although not directly involved with oculomotor tracking, the cleidomastoid and trapezius play a receptive role in stabilizing the head and upper limb [40, 76]. Finally, since the accessory nerve originally evolved having a purely motor efferent output, it makes sense that an anastomosis must occur with upper cervical spinal nerve to attain proprioceptive afferents for the target musculature. The accessory nerve often anastomoses directly with the dorsal root ganglion of the first spinal nerve as noted by many authors [23, 76, 92, 94, 98]. In spite of the obvious connection with the upper cervical nerves, the accessory nerve in some mammals retains a fundamental link to its cranial origins. In both the rat and the cat, the spinal accessory conveys axons to the vagus nerve strongly supporting its vagal origin [107–109]. Although tedious, there is a logical explanation for the behavior of the accessory nerve. Truly, one of the marvels of comparative anatomy, the accessory nerve has evolved into a nerve that is not defined under our traditional cranial or spinal categories, and thus prompts a new class of nerve, the transitional nerve.

## 6. Summary

1. A thorough review of historical anatomical writings indicates the direct translation of early Greek or Latin to be “encephalic”, opposed to “cranial” as the descriptor for nerves originating within the brain or brainstem. We propose using the term “encephalic” to describe any nerve originating within the brain or brainstem.

(2) The accessory nerve was not classified as a cranial nerve by Thomas Willis, who is credited with the first accurate description of the nerve in humans. Willis refers to the accessory nerve as an irregular spinal nerve, which supports our proposal that the accessory nerve is not of cranial origin.

(3) The “cranial” portion of the accessory was likely adopted by Fredrici Arnold and has been strongly refuted by studies in the fields of comparative anatomy, topical anatomy, molecular biology, and embryology. We propose renaming the “cranial” accessory nerve to laryngopalatopharyngeal nerve and maintain that it is the only structure to represent the eleventh cranial nerve in today's current cranial nerve classification. However, the author's do not support the current cranial nerve classification. Suggested renumeration of the cranial nerves (Table 2).

(4) Embryologic investigations have shown that muscles of the neck region do not develop under the same guiding principles as the head and neck. The SCM and Trapezius have mesoderm-derived striated muscle with connective tissue and osseous attachments that are neural crest born. Furthermore, the SCM and Trapezius are innervated by a nerve that itself is transitional in nature, having both cranial and spinal characteristics, but ultimately residing in the cervical spinal cord. Therefore, the accessory nerve and its associated musculature should be regarded as a *transitional nerve and transitional muscles*, a new category of peripheral nerve and musculature.

(5) By observing phylogenetic trends, the development of the accessory nerve can be explained under the principles of neurobiotaxis.

## Figures and Tables

**Table 1:** Classification of the efferent peripheral nerves innervating striated musculature based on embryological and anatomical findings. All cranial nerves have a nucleus within the brainstem and receive mesenchymal contributions from neural crest cells. The cranial nerves can be subdivided into 3 groups. CSEpr cranial nerves innervate striated muscle derived from preotic somites. CSEpo cranial nerves innervate striated musculature from postotic somites, while CBE cranial nerves innervate striated muscle developing from somitomeres (head mesoderm). Spinal nerves have a nucleus within the spinal cord and muscles of somitic origin. Trunk Mesenchyme also forms from the mesodermal layer. The transitional nerve (accessory proper) has a nucleus within the spinal cord, innervates muscle derived from somites, and has mesenchymal elements that form from neural crest cells, thus making it a unique peripheral nerve. It is important to note that the eleventh crania nerve is represented entirely by the laryngopalatopharyngeal motor (LPP) formerly the cranial root of the accessory.

	Target muscle(s)	Nucleus location	Mesoderm	Neural crest	Classisfication
Cranial Nerves					
III	Extraocular eye m.'s	Brainstem	Preotic Somite	YES	CSE_pr_
IV	Superior Oblique m	Brainstem	Preotic Somite	YES	CSE_pr_
V	Muscles of Mastication	Brainstem	L.P.M.	YES	CBE
VI	Lateral Rectus m	Brainstem	Preotic Somite	YES	CSE_pr_
VII	Facial Expression/2nd Jaw	Brainstem	L.P.M.	YES	CBE
IX	Pharynx	Brainstem	L.P.M.	YES	CBE
X	Int Laryn. Palate, Pharynx	Brainstem	Occipital Somites	YES	CSE_po_
XI-Palatopharyngeal	Pharynx	Brainstem	Occipital Somites	YES	CSE_po_
XII	Tongue m.'s	Brainstem	Occipital Somites	YES	CSE_po_

Transitional neve					
XI-accessory proper	SCM and trapezius	Spinal cord	Cervical somites	YES	TSE

Spinal Nerves					
31 human	Respective myotome	Spinal cord	Somite	NO	GSE

**Table 2:** Renumeration of cranial nerves following the application of the definition of a cranial nerve. To be defined as a cranial nerve the nuclei must originate from the brainstem, communicate with a foramen of the skull and secondary neuron whose cell bodies are located in the brainstem. This criteria still produced 12 cranial nerves.

Current order	Assessment results	New order
(1) Olfactory	Eliminates, (1) nucleus not in brainstem, (2) primary sensory neuron	
(2) Optic	Eliminated, (1) nucleus not in brainstem, (2) primary sensory neuron	
(3) Oculomotor	Becomes 1st cranial nerve	(1) Oculomotor
(4) Trochlear	Becomes 2nd cranial nerve	(2) Trochlear
(5) Trigeminal	Is split into 2 separate nerves due to separate nuclei-current sensory remains as trigeminal with ophthalmic, maxillary, and mandibular divisions as 4th cranial nerve, motor of trigeminal becomes the masticatory nerve and is now the 3rd cranial nerve	(3) Masticatory (4) Trigeminal
(6) Abducens	Moves to the 5th cranial nerve	(5) Abducens
(7) Facial	Due to separate nuclei, facial becomes 6th cranial nerve; nervous intermedius becomes the 7th cranial nerve	(6) Facial (7) Nervous Intermedius
(8) Vestibulocochlear	Is split into 2 nerves due to separate nuclei and separate modalities. Vestibular nerve becomes the 8th cranial nerve; and the cochlear nerve becomes the 9th cranial nerve	(8) Vestibular (9) Cochlear
(9) Glossopharyngeal	Becomes the 10th cranial nerve	(10) Glossopharyngeal
(10) Vagus	Is split into 2 divisions due to target organs:	(11) Vagus:
	(1) Laryngopalatopharyngeal (formerly cranial root of 11)	(a) Laryngopalatopharyngeal
	(2) Thoracoabdominal	(b) Thoracoabdominal
(11) Accessory	Eliminated, nucleus not in brainstem	
(12) Hypoglossal	Remains the same	(12) Hypoglossal
